# Autoimmune hemolytic anemia and thrombocytopenia in a Chinese patient with heterozygous NBAS mutations: Case report

**DOI:** 10.1097/MD.0000000000036975

**Published:** 2024-03-22

**Authors:** Yuanlin Yang, Xiaoming Fei, Fang Lei, Lixia Wang, Xianqiu Yu, Yu Tang

**Affiliations:** a Department of Hematology, Affiliated Hospital of Jiangsu University, Zhenjiang, China; b Department of Rheumatology, Affiliated Hospital of Jiangsu University, Zhenjiang, China.

**Keywords:** ANKRD26, autoimmune hemolytic anemia, NBAS, thrombocytopenia

## Abstract

**Rationale:**

Neuroblastoma amplified sequence (NBAS)-associated disease is an autosomal recessive disorder and a broad spectrum of clinical symptoms has been reported. However, autoimmune mediated hemolytic anemia (AIHA) is rarely reported in NBAS disease.

**Patient concerns:**

A now 21-year-old male harbors heterozygous variants of c.6840G>A and c.335 + 1G>A and was found had retarded growth, hypogammaglobulinemia, B lymphopenia, optic atrophy, horizontal nystagmus, slight splenomegaly and hepatomegaly since childhood. This case had normal hemoglobin level and platelet count in his childhood. He developed AIHA first in his adulthood and then thrombocytopenia during the treatment of AIHA. The mechanism underlying a case with pronounced hypogammaglobulinemia and B lymphopenia is elusive. In addition to biallelic NBAS mutations, a germline mutation in the ANKRD26 (c.2356C>T) gene was also detected. So either autoimmune or ANKRD26 mutation-mediated thrombocytopenia is possible in this case.

**Intervention and outcome:**

He was initially managed with steroid and intermittent intravenous immunoglobulin supplement. After treatment, he responded well with a normalization of hemoglobin and serum bilirubin. But the patient subsequently experienced severe thrombocytopenia in addition to AIHA. He was then given daily avatrombopag in addition to steroid escalation. He responded again to new treatment, with the hemoglobin levels and platelet counts went back to the normal ranges. Now he was on de-escalated weekly avatrombopag and low-dose steroids maintenance.

**Conclusion:**

The phenotype of this case indicates that c.335 + 1G>A NBAS variant is probably a pathogenic one and c.2356C>T ANKRD26 variant is improbably a pathogenic one. AIHA may respond well to steroid even when happened in patients with NBAS disease.

## 1. Introduction

Neuroblastoma amplified sequence (NBAS) previously defined as a co-amplified gene with the N-myc gene in neuroblastoma, it contains 52 exons and localized on chromosome 2p24 nearest to N-myc.^[1,2]^ NBAS can act together with the nonsense-mediated mRNA decay pathway to regulate a large number of endogenous RNA targets throughout the evolutionary process, depletion of NBAS genes alters the expression of 1444 genes.^[3]^ Pathogenic mutations in the NBAS gene cause reduced protein expression and NBAS-associated disease is a rare autosomal recessive disorder.^[4]^ Biallelic pathogenic variants in NBAS were first reported in Yakuts with SOPH syndrome (short stature, optic atrophy, and Pelger-Huët anomaly),^[5]^ subsequently, mutations in the NBAS gene were found to be closely associated with fever-related recurrent acute liver failure (ALF).^[6]^ Currently, it had reported a broad spectrum of clinical symptoms that can involve the liver, growth, skeletal system, nervous system, epidermis, immune system, and muscles in NBAS-associated disease.^[7–9]^ Hematologic manifestations of patients with NBAS mutations include neutropenia, lymphopenia, Pelger-Huët anomaly, reduced natural killer cells, low numbers of CD8+ T cells and high CD4+/CD8+ T cell-ratios,^[4]^ however, autoimmune hemolytic anemia (AIHA) was rarely reported in NBAS. Besides, how to manage the AIHA in patients with NBAS mutations is currently unknown.

Here, we describe a case of a young adult male with compound heterozygous NBAS variants, who developed AIHA in his adulthood. During the follow-up of this case, he subsequently had thrombocytopenia in addition to AIHA, which fulfills the diagnostic criteria for Evans syndrome. The hemolytic anemia and thrombocytopenia in this case was successfully managed with corticosteroid, avatrombopag and intravenous immunoglobulin. This report was approved by the Affiliated Hospital of Jiangsu University Review Board, in accordance with the Declaration of Helsinki. The patient has provided informed consent for publication of this case.

## 2. Case presentation

**Figure 1.**
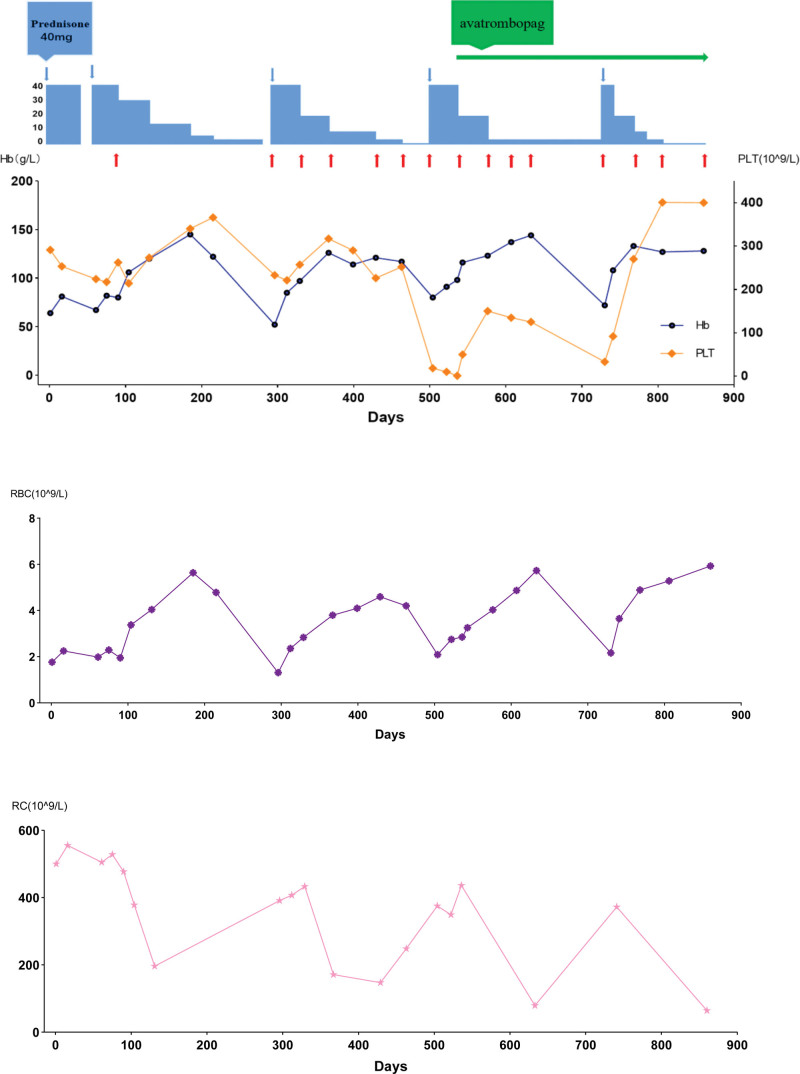
Genetic trait of his family.

**Figure 2.**
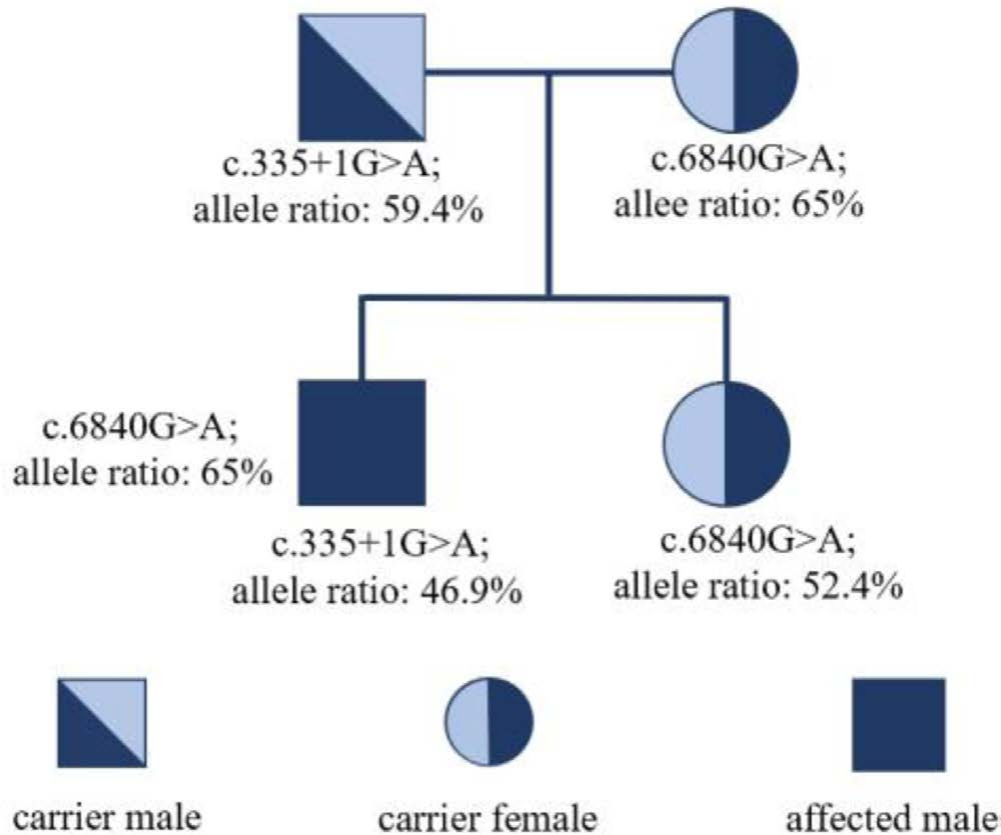
The medications this patient received and the changes in blood parameters. Prednisone is represented by a blue bar, the height of which represents the drug dose, red arrows represent Intravenous immunoglobulin, green arrow represent avatrombopag. Hb = hemoglobin, PLT = blood platelets, RBC = red blood cells, RC = reticulocytes.

This is a male, 21 year old, presented with retarded growth, hypogammaglobulinemia, optic atrophy, horizontal nystagmus, slight splenomegaly and hepatomegaly since childhood. He had only intermittent slightly elevated liver transaminases which were asymptomatic and no episode of acute liver failure in his life. He has normal intelligence and blood counts were normal during the whole childhood. This case had received recombinant growth factor therapy when he was a child, but it had little help with his short stature. He visited our institute for anemia and jaundice in 2019. In his diagnostic workup, it showed elevated peripheral blood reticulocyte count, Lactate dehydrogenase, indirect bilirubin and free hemoglobin, the direct Coombs’ test was positive (both C3 and IgG), which was consistent with autoimmune mediated hemolytic anemia (AIHA). Interestingly, he had serum IgG 2g/L (reference range 7.5–14.5), undetectable IgA and IgM. Flow cytometry of peripheral blood lymphocyte subsets showed 91% of T-lymphocytes, 8% of NK cells and only 1% of B-lymphocytes. Peripheral blood smear showed no increase of blast or any Pelger-Huët anomaly. Bone marrow examination showed no increase of blast or signs of myelodysplasia. Karyotype examination showed 46 XY, 21p+[12]/46 XY [3]. He was initially managed with steroid and intermittent intravenous immunoglobulin supplement. After treatment, he responded well with a normalization of hemoglobin and serum bilirubin. Then we did whole genome sequencing of his family, it found that his mother had NBAS mutation (c.6840G>A; allele ratio: 65%), ANKRD26 mutation (c.2356C>T; allele ratio: 34.5%), PALB2 mutation (c.1213C>G; allele ratio: 54.2%). His father had NBAS mutation (c.335 + 1G>A; allele ratio: 59.4%). This case had all of above-mentioned mutations with similar allele ratios. His sister had only NBAS mutation (c.6840G>A; allele ratio: 52.4%) (see Fig. 1). All his parents and sister are healthy and have normal blood counts. In October 2021, he had a newly developed thrombocytopenia in addition to anemia. There was no abnormal finding in the antinuclear antibody panel. He was then given daily avatrombopag in addition to steroid escalation. He responded again to new treatment, with the hemoglobin levels and platelet counts went back to the normal ranges. Now he was on de-escalated weekly avatrombopag and low-dose steroid maintenance. During the follow-up of this case, his hemoglobin levels, platelet counts and treatment adjustment are shown in Figure 2.

## 3. Discussion

Since the first report of NBAS-associated disease in 2010, more cases or case series have been subsequently published in literature or reported at conferences. Current research indicates that NBAS-related disorders are autosomal recessive disorders with a wide range of clinical symptoms that can involve the liver, growth, skeletal system, nervous system, epidermis, immune system, and muscles.^[7–9]^ In a study with the largest cohort of 110 patients, the main organ systems or processes involved in NBAS-associated diseases were liver (97%), growth (74%), skeletal system (73%), nervous system (69%), integument (67%), immune system (61%), and musculature (53%).^[4]^ In another report analyzing 14 newly diagnosed Chinese patients, all patients had liver involvement, 9 patients had extrahepatic phenotypes including skeletal involvement (4/12), growth failure (3/11), intellectual disability (3/14), hypoimmunoglobulin (3/14), ophthalmic abnormalities (2/14), facial dysmorphism (1/13), and cardiac abnormalities (1/14).^[10]^

What’s peculiar in this case is the AIHA developed in his adulthood. AIHA is a decompensated acquired hemolysis caused by the host’s immune system acting against its own red cell antigens. The essence of AIHA is that it is caused by the increased destruction of erythrocytes by anti–erythrocyte autoantibodies. Since there is significant deficiency of both B-lymphocytes and immunoglobulins in this case, it makes it is hard for us to understand the emergence of anti–erythrocyte autoantibodies. One possible explanation is the dysregulation of the central and peripheral self-tolerance and the development of autoreactive B lymphocytes from the residual B cells. Currently, there is only one AIHA case has been reported in NBAS-associated disease, so it is unlikely that the AIHA is within the spectrum of multisystemic manifestations result from NBAS mutations.^[11]^ No matter this case is an idiopathic or not, his AIHA responded well to corticosteroid. However, it seemed that the AIHA is not a self-limited one in this case, since the hemolytic episodes recurred several times during steroid tapering.

There are a total 1194 variants reported in ClinVar Miner(Version 2022-10-30), and large number of variants are thought to be pathogenic. A total number of 86 different variants were reported by Staufner et al,^[4]^ which including missense, frameshift, splice site, nonsense, exon deletions, in-frame deletions and intronic variant. There are 66 genotypes, 13 of them homozygous. In the current case, he inherits a c.6840G>A variant from his mother, which has not been previously reported in a total of 24 Chinese case cohort.^[10]^ The effect of the c.6840G>A substitution altered NBAS transcript splicing leading to skipping of exon 51 and, in turn, to a mature messenger RNA (mRNA) variant encoding for a protein that lacked an in-frame portion of 43 amino acid residues within the C-terminal region.^[4]^ On the contrary, it’s less clear the effect of c.335 + 1G>A variant on the transcription or translation of NBAS and this variant is listed as likely pathogenic one in ClinVar database (Accession: VCV000801651.1). Based on the clinical manifestations of this case with a heterozygote of c.6840G>A and c.335 + 1G>A, c.335 + 1G>A variant should be changed into a “pathogenic” variant category.

Based on the regions of NBAS protein the variants affect, NBAS-associated diseases can be classified into 3 clinical subgroups: β-propeller (combined ALF and multisystemic phenotype), Sec39 (mainly ALF) and C-terminal (predominant multisystemic phenotype).^[4]^ Because this case has a heterozygote of c.6840G>A and c.335 + 1G>A, so it is supposed to involve both β-propeller and C-terminal of NBAS protein, which may explain the reason of development of AIHA along with other multisystemic symptoms. However, another case of NBAS mutation patient also presented with AIHA had a different c.5741G>A and c.6433-2A>G heterozygote, both variants locate in the C-terminal of NBAS.^[11]^ From those 2 cases with AIHA manifestation, we cannot find any correlation between regions of NBAS variant and AIHA.

After the initial clinical manifestation of AIHA, this case subsequently developed thrombocytopenia, which makes it meet the diagnostic criteria for Evan’s syndrome.^[12]^ However, the presence of ANKRD26 variant (c.2356C>T) challenges a mechanism of autoimmune-mediated thrombocytopenia. ANKRD26-related thrombocytopenia (ANKRD26-RT) is an autosomal dominant, non-syndromic inherited thrombocytopenia with normal platelet size and predisposition to myeloid neoplasms.^[13]^ Currently, vast majority of the causative variants were detected in a 19-nucleotide region of the 5′untranslated region (UTR)(c.-116 through c.-134).^[13,14]^ Besides, the penetrance of the thrombocytopenic trait in patients with ANKRD26-RT is complete and degree of thrombocytopenia variable, but typically it is mild to moderate.^[14]^

This case had no thrombocytopenia during his childhood and his mother has a normal platelet count. So we think ANKRD26-RT is highly likely not to be cause of thrombocytopenia in this case. Although ANKRD26:c.2356C>T (Variation ID: 1703797) is listed as a pathogenic variant in ClinVar database, based on the clinical features of this case, c.2356C>T variant may not be suggested to be a pathogenic one. If the cause of thrombocytopenia is ANKRD26-RT, an important principle in the management is avoiding harm by preventing patients from receiving treatments such as immunosuppressive treatment and splenectomy.^[13]^ We added thrombopoietin receptor agonist (TPO-RA) avatrombopag to combine with steroid, and this case responded well to TPO-RA with a normalization of platelet count.

## 4. Conclusion

We reported a case with heterozygote of c.6840G>A and c.335 + 1G>A NBAS variants who developed AIHA, and highly likely Evan’s syndrome in his adulthood in addition to other multisystemic symptoms. The mechanisms of the development of antibody-mediated autoimmune disease in a subject with B-lymphocyte deficiency is elusive and deserve further investigation. Based on the clinical manifestations and genetic trait of his family, c.335 + 1G>A NBAS variant is suggested to be a pathogenic one instead of likely pathogenic one, c.2356C>T ANKRD26 variant may be changed to nonpathogenic variant from pathogenic one.

## Author contributions

**Supervision:** Xiaoming Fei, Fang Lei, Lixia Wang, Xianqiu Yu, Yu Tang.

**Writing – original draft:** Yuanlin Yang.

**Writing – review & editing:** Xiaoming Fei.
